# Preservation of neurocognitive function and local control of 1 to 3 brain metastases treated with surgery and carmustine wafers

**DOI:** 10.1002/cncr.28307

**Published:** 2013-08-23

**Authors:** Steven Brem, Christina A Meyers, Gary Palmer, Margaret Booth-Jones, Surbhi Jain, Matthew G Ewend

**Affiliations:** 1 Department of Neuro-Oncology, Moffitt Cancer CenterTampa, Florida; 2 Department of Neurosurgery, Perelman School of Medicine, University of Pennsylvania and the Abramson Cancer CenterPhiladelphia, Pennsylvania; 3 Department of Neuro-Oncology, The University of Texas MD Anderson Cancer CenterHouston, Texas; 4 Department of Medical Affairs, Eisai Inc.Woodcliff Lakes, New Jersey; 5 Department of Neurosurgery and the Lineberger Comprehensive Cancer Center, University of North CarolinaChapel Hill, North Carolina

**Keywords:** brain metastases, carmustine wafers, neurocognitive function, stereotactic radiosurgery, whole brain radiotherapy

## Abstract

**Background:**

Neurosurgical resection and whole-brain radiation therapy (WBRT) are accepted treatments for single and oligometastatic cancer to the brain. To avoid the decline in neurocognitive function (NCF) linked to WBRT, the authors conducted a prospective, multicenter, phase 2 study to determine whether surgery and carmustine wafers (CW), while deferring WBRT, could preserve NCF and achieve local control (LC).

**Methods:**

NCF and LC were measured in 59 patients who underwent resection and received CW for a single (83%) or dominant (oligometastatic, 2 to 3 lesions) metastasis and received stereotactic radiosurgery (SRS) for tiny nodules not treated with resection plus CW. Preservation of NCF was defined as an improvement or a decline ≤1 standard deviation from baseline in 3 domains: memory, executive function, and fine motor skills, evaluated at 2-month intervals.

**Results:**

Significant improvements in executive function and memory occurred throughout the 1-year follow-up. Preservation or improvement of NCF occurred in all 3 domains for the majority of patients at each of the 2-month intervals. NCF declined in only 1 patient. The chemowafers were well tolerated, and serious adverse events were reversible. There was local recurrence in 28% of the patients at 1-year follow-up.

**Conclusions:**

Patients with brain metastases had improvements in their cognitive trajectory, especially memory and executive function, after treatment with resection plus CW. The rate of LC (78%) was comparable to historic rates of surgery with WBRT and superior to reports of WBRT alone. For patients who undergo resection for symptomatic or large-volume metastasis or for tissue diagnosis, the addition of CW can be considered as an option.

## Introduction

Brain metastases affect 25% to 45% of patients with cancer.^1^–^3^ The rising incidence of metastasis to the brain threatens to limit gains made by new systemic treatments.^4^ Both the blood-tumor barrier and tumor-stromal interactions impede the delivery of systemic drugs to tumors in the brain.^3^–^5^

Based on the trials of Patchell and colleagues,^6^,^7^ neurosurgical resection and whole-brain radiation therapy (WBRT) are mainstays of current therapy.^8^–^11^ WBRT helps prevent local recurrence and new distant brain metastases,^7^,^12^–^14^ but it fails to improve the duration of functional independence or overall survival.^13^,^14^ WBRT carries risks of neurocognitive impairment, leukoencephalopathy, and brain atrophy.^15^–^17^ Thus, as patients with brain metastases live longer, increasing concern regarding neurocognitive function (NCF) has led to a paradigm shift to defer WBRT and treat locally with surgery and/or stereotactic radiosurgery (SRS) while monitoring for distant relapses.^12^,^14^,^18^,^19^

To enhance drug delivery, biodegradable carmustine polymer wafers (Gliadel; Eisai, Inc., Woodcliff Lake, NJ) were developed to be implanted into the surgical cavity of malignant gliomas.^20^–^22^ Preclinical, orthotopic models demonstrated prolonged survival and local control (LC) when carmustine wafers were used to treat brain metastases.^23^,^24^ In the only published clinical trial that used carmustine wafers to treat brain metastases, we demonstrated excellent LC when carmustine wafers were combined with resection and WBRT.^25^ Given the known NCF risks linked to WBRT,^12^,^17^–^19^ we designed the current study to evaluate the LC rate with resection and chemowafers while deferring WBRT. We hypothesized that NCF would be preserved or improved as we monitored the “cognitive trajectory” after surgery.^26^

## Materials and Methods

This prospective, single-arm, phase 2 trial (NCT00525590) involved 12 US centers between December 2007 and December 2010. The institutional review board at each site approved the protocol, following Good Clinical Practice guidelines and the Declaration of Helsinki. All patients provided informed consent. A data safety committee monitored the study.

Eligible patients had 1 to 3 brain metastases, including patients with a single resectable metastasis or with 2 metastases that were resectable as 1 cavity (distance apart, <1 cm); age >18 years; a Karnofsky performance status (KPS) ≥70; and a recursive partitioning analysis (RPA) classification of 1 (35%) or 2 (65%).^1^,^27^ The most common histology (41%) was nonsmall cell lung cancer. Exclusion criteria included: small cell lung cancer, thyroid cancer, lymphoma, germ cell cancer, or primary central nervous system (CNS) tumor; prior cranial irradiation or exposure to carmustine; leptomeningeal disease; surgically targeted metastasis in brainstem or posterior fossa; pregnancy; unstable medical or psychiatric status; and receipt of investigational drugs or bevacizumab within 30 days or of anticoagulants within 7 days of entry.

Initially, the protocol included only patients with a single cerebral metastasis, based on the assumption that half of all metastases are single and, thus, are potentially treatable by surgical resection.^6^ However, with modern high-resolution magnetic resonance imaging (MRI) scans, additional asymptomatic micrometastases that are amenable to radiosurgery can be detected in up to 40% of patients.^28^ Therefore, the protocol was amended to include patients who had 1 to 3 MRI-detectable metastases for whom surgery was indicated for a dominant or symptomatic tumor, and SRS was an option for all remaining lesions. WBRT was withheld until patients developed recurrent disease.

**Figure 1:**
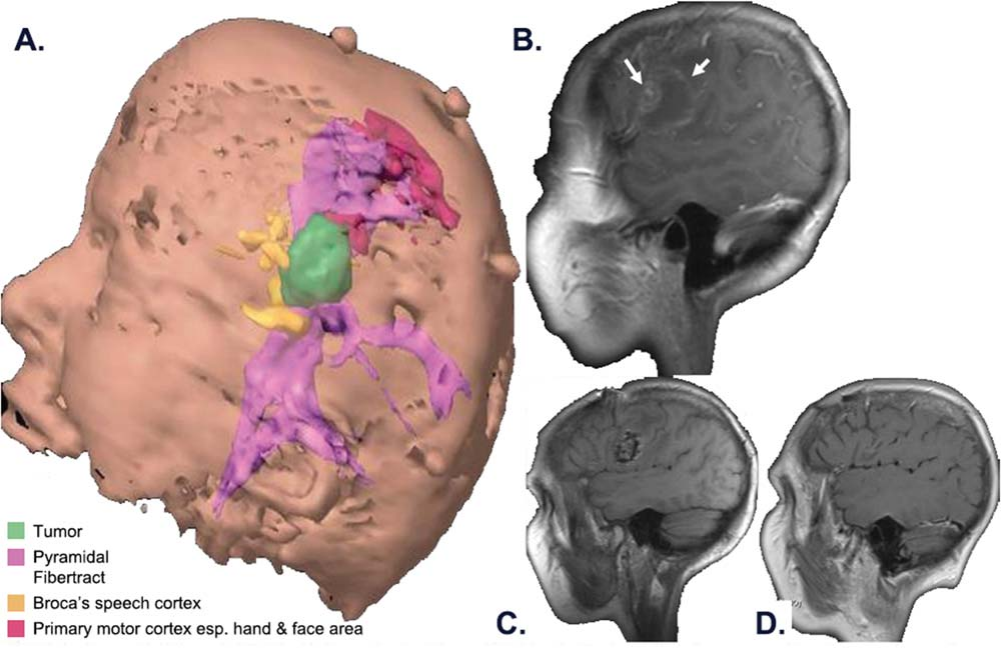
Preoperative and postoperative magnetic resonance images (MRIs) are shown. A patient with metastatic melanoma developed aphasia and facial weakness. (A) The 3-dimensional preoperative model reveals the tumor (green), the primary motor cortex (red) and arcuate fasciculus (purple), the Broca speech area (yellow), and the corticospinal tract (purple). (B) This preoperative sagittal MRI reveals a single hemorrhagic metastasis (arrows) in the left frontal lobe near the primary motor and the Broca speech areas. (C) This postoperative MRI reveals resection of the tumor and intracavitary placement of the carmustine wafers. Speech, motor, and neurocognitive function returned postoperatively. (D) A follow-up scan shows no tumor recurrence at 34 months.

All patients underwent resection of a dominant or single metastasis. Surgery was regarded as the best treatment option considering 1 or more of the following criteria: 1) tumor volume, 2) symptoms from mass effect or edema, or 3) need for histologic diagnosis. Advanced surgical techniques—including image guidance, microsurgery, tractography, and brain mapping—achieved gross total resection while minimizing the risk to surrounding brain (Fig. 1).

After metastasis resection and histologic confirmation by frozen section, 1 to 8 carmustine wafers were used to line the tumor cavity. Water-tight dural closure, known to decrease complications,^25^ was used routinely. Ventricular openings <1 cm^2^ were sealed with resorbable hemostatic material.^21^ Chemowafers were avoided in the subarachnoid space, especially if the neurosurgeon encountered a major cerebral artery or infiltration of tumor. Corticosteroids and anticonvulsants were prescribed judiciously by the treating physician.^21^,^25^

Within 14 days before surgery, patients underwent baseline assessment, including history, physical examination, MRI, NCF, activities of daily living (ADL), KPS, and RPA classification.^27^ A list of concurrent medications was maintained. An MRI was obtained within 48 hours after surgery; then, follow-up MRI, NCF, ADL, KPS, RPA classification, and toxicity assessments were obtained at months 2, 4, 6, 9, and 12. Patients were followed for 12 months unless there was evidence of treatment failure (eg, local recurrence), neurologic decline because of CNS metastases, withdrawal from the study, or death.

The NCF assessment comprised 3 domains: 1) *memory* using the revised Hopkins Verbal Learning Test, 2) *executive function* using the Controlled Oral Word Association and Trailmaking Test B, and 3) *fine motor coordination* using the pegboard dominant and nondominant hand tests.^29^,^30^

Postsurgical imaging studies were reviewed for evidence of CNS recurrence.^25^ Local recurrences were defined as within 2 cm of the initial resection cavity. Distant CNS recurrences were defined as any of the following: >2 cm from the initial surgical site; or the contralateral side; or in the posterior fossa, spinal cord, or cerebrospinal fluid (carcinomatous meningitis). Adverse events (AEs) were graded for severity (mild, moderate, severe, or fatal) and attribution (unlikely, possibly, probably, or definitely related to CW use). Toxicities were graded using National Cancer Institute Common Toxicity Criteria, version 2.0.^25^

The trial design was based on statistical assumptions: a 35% rate of neurocognitive decline within 12 months, a 20% rate of local recurrence within 12 months, and a 15% drop-out rate. Accordingly, 50 to 58 patients were required for a 2-sided 95% confidence interval (CI).

NCF endpoints included standardized score changes (from baseline) for each visit, including time and severity of neurocognitive change. Each NCF domain had an average standardized Z-score, and changes from each patient’s own baseline were analyzed using the *t* test for paired data (with 95% CIs). Preservation of NCF was defined as a decrease of ≤1 standard deviation (SD), any improvement, or no change (0 SD); and worsening was defined as a decrease >1 SD. Deterioration in NCF was defined as deterioration from baseline in at least 2 domains. For each domain, deterioration occurred if a Z-score average decreased from baseline by ≥3 SD on 2 consecutive visits or at the last follow-up visit. For a missing NCF evaluation, if the reason was “neurologic,” then a score of −5.00 was imputed.

Other endpoints included the rates of local and distal tumor recurrence; the time to recurrence; and the correlation of recurrence with mass effect, cognitive functioning, and clinical symptoms. The time to recurrence was analyzed using the Kaplan-Meier method.^31^ Univariate and multivariate Cox proportional hazard models for the time to local, distant, and overall recurrence were produced using 11 predictive factors (baseline tumor volume, age, RPA classification, KPS, NCF score, resected tumor volume, number of implanted wafers, number of metastatic tumors, primary cancer type, and radiation sensitivity).

## Results

**Figure 2:**
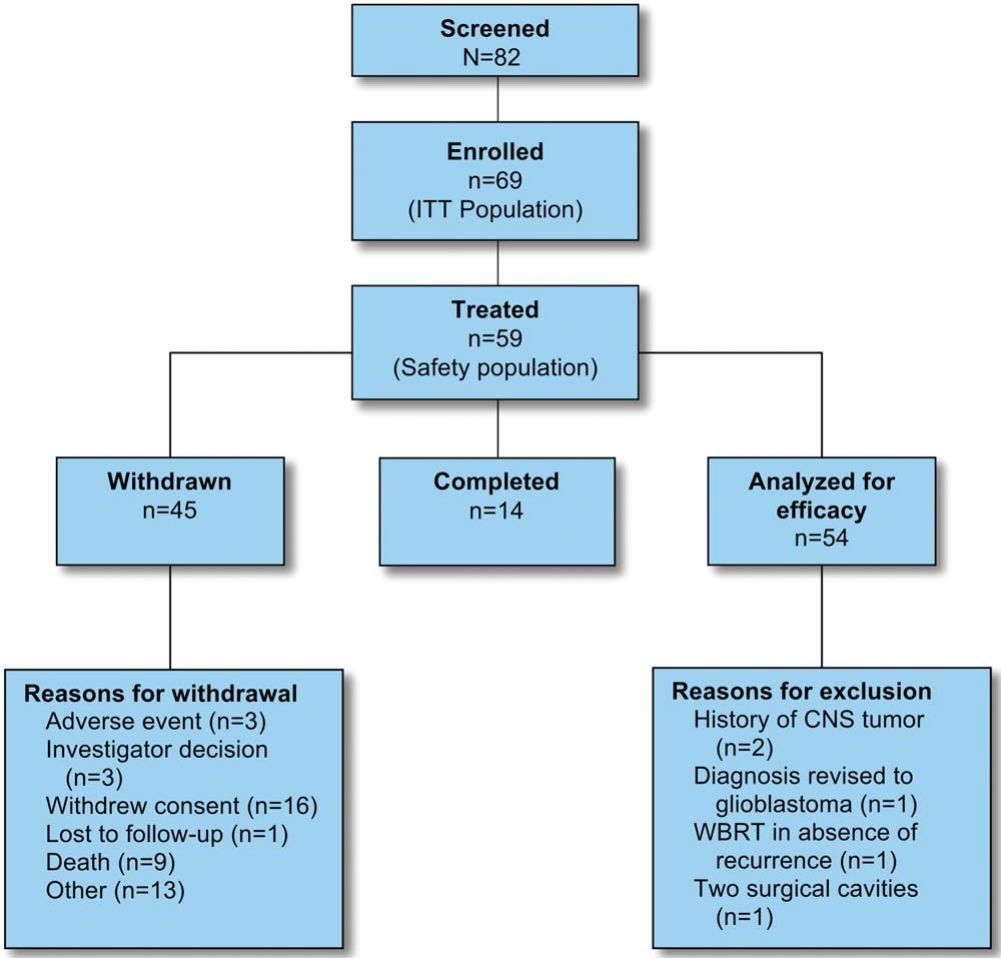
Participant flow is illustrated. CNS indicates central nervous system; WBRT, whole-brain radiation therapy.

Eighty-two patients were screened, 69 were enrolled, and 59 received treatment (Fig. 2, Table 1). Patients were followed for a median of 7.7 months. Fourteen of 59 patients (24%) completed the 12-month assessment period; 5 (36%) initially had an RPA classification of 1, and 9 (64%) initially had an RPA classification of 2. Five patients were excluded from efficacy analyses because of protocol violations after wafer implantation.

**Table 1:** Patient Demographics and Cancer History: Safety Population (n = 59)

Variable	No. of Patients (%)
Sex	
Men	26 (44)
Women	33 (56)
Age: Median [range], y	63 [37–81]
Race	
White	55 (93)
Black or African American	1 (2)
Asian	2 (3)
American Indian or Alaska Native	1 (2)
RPA classification (n = 54)	
Class 1	19 (35)
Class 2	35 (65)
Type of primary cancer	
NSCLC	24 (41)
Melanoma	15 (25)
Breast	7 (12)
Renal	4 (7)
Other	7 (12)
Unknown	2 (3)
Primary cancer status	
Newly diagnosed, no prior therapy	18 (31)
Previously diagnosed, off therapy ≥30 d	36 (61)
Previously diagnosed, receiving systemic chemotherapy	5 (8)
No. of brain lesions on MRI	
1	49 (83)
2	6 (10)
3	4 (7)

Abbreviations: MRI, magnetic resonance imaging; NSCLC, non–small cell lung cancer; RPA, recursive partitioning analysis.

Preservation or improvement of NCF was observed in all 3 domains for the majority of patients throughout the study (month 2, 26 of 46 patients [57%]; month 4, 24 of 37 patients [65%]; month 6, 17 of 26 patients [65%]; month 9, 14 of 22 patients [64%]; and month 12, 9 of 14 patients [64%]). In 1 patient (2%), NCF deterioration was observed in the fine motor coordination domain at months 2 and 4 and in the memory domain at month 4 (last follow-up visit).

**Figure 3:**
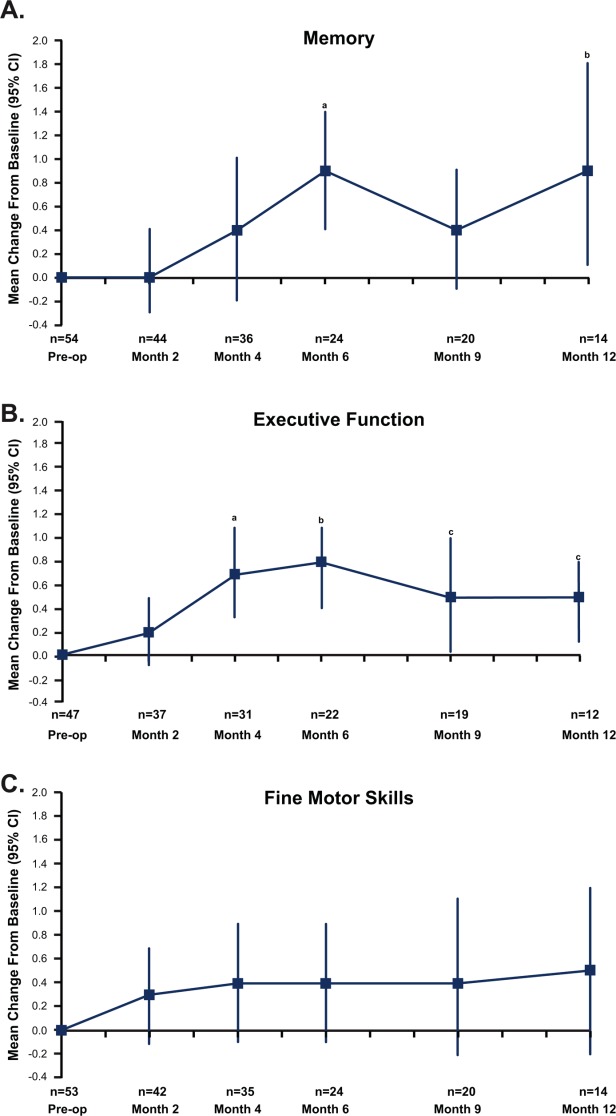
Improvement in neurocognitive function was measured after surgical resection and carmustine wafer placement for brain metastasis using average, standardized Z-scores (indicated as the mean change with 95% confidence interval [CI]). Statistically significant changes were noted in (A) memory (a, *P* = .001; b, *P* = .029) and (B) executive function (a, *P* = .001; b, *P* = .0007; c [left], *P* = .041; c [right], *P* = .018). (C) Fine motor skills.

For the individual domains, the cumulative rates of preservation and worsening of NCF were determined. In the memory domain, 37 of 54 patients (69%) had preservation of NCF (including 26 of 54 patients [48%] who had improvement), 8 of 54 patients (15%) had worsening NCF, and 9 patients were not assessed. In the executive function domain, 39 of 54 patients (72%) had preservation of NCF (including 23 of 54 patients [43%] who had improvement), 4 of 54 patients (7%) had worsening NCF, and 11 patients were not assessed. In the fine motor coordination domain, 34 of 54 patients (63%) had preservation of NCF (including 27 of 54 patients [50%] who had improvement), 11 of 54 patients (20%) had worsening NCF, and 9 patients were not assessed. Mean changes in Z-scores are provided in Figure 3.

**Figure 4:**
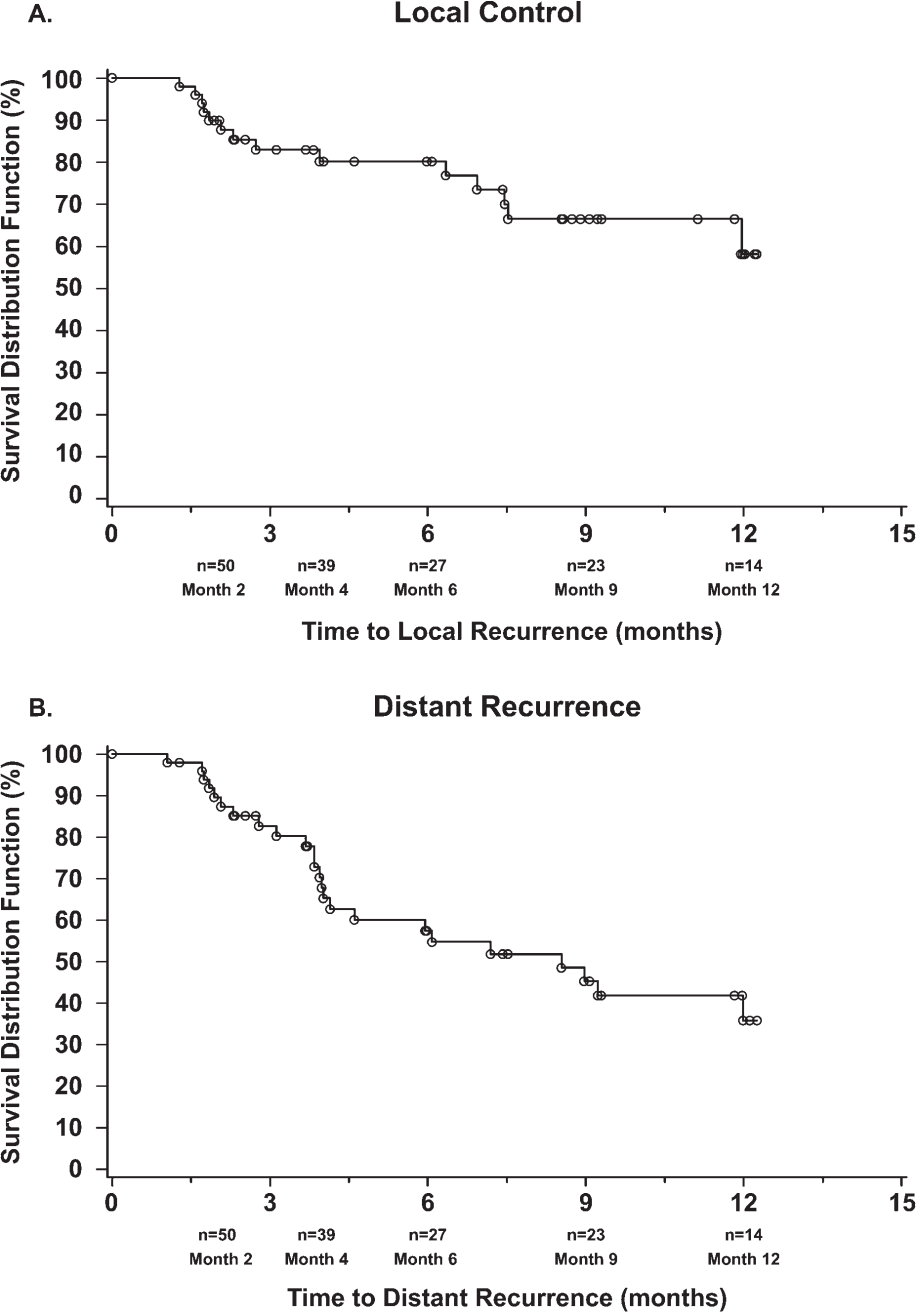
These are Kaplan-Meier curves for (A) local recurrence and (B) distant recurrence. The median time to distant recurrence was 8.5 months.

Fifty of our 54 patients had postbaseline MRI assessments. Local recurrences (Fig. 4A) developed in 14 of 50 evaluable patients (28%). The recurrence rate varied with histology: patients with melanoma had the lowest recurrence rate (7%; 1 of 15 patients), those with nonsmall cell lung cancer had a moderate rate (24%; 5 of 21 patients), and patients with breast cancer had the highest rate (57%; 4 of 7 patients). These differences were not statistically significant. Univariate predictors of the time to local recurrence were: 1) baseline total tumor volume (hazard ratio [HR], 1.043; *P* = .009) and 2) the number of wafers implanted (HR, 1.366; *P* = .040), which is also a function of tumor volume. Significant multivariate predictors of the time to local recurrence were: 1) baseline total tumor volume (HR, 1.095; *P* = .001), 2) primary cancer status (previously diagnosed and receiving systemic chemotherapy vs newly diagnosed and no prior therapy; HR, 22.506; *P* = .013), 3) baseline KPS (HR, 0.922; *P* = .031), and 4) baseline NCF score (HR, 1.534; *P* = .002).

Distant recurrences (Fig. 4B) developed in 24 of 50 patients (48%) and consisted of diverse histologies, including nonsmall cell lung cancer (33%; 7 of 21 patients), breast cancer (57%; 4 of 7 patients), and melanoma (53%; 8 of 15 patients). Over half of the distant recurrences (17 of 24 recurrences) developed by month 4. Recurrences in these patients were treated with SRS or WBRT, and they achieved favorable oncologic control without neurologic-related death.

The median number of wafers inserted was 5 (range, 1.5-8.0 wafers), corresponding to a dose of 38.5 mg carmustine. Eight patients with large tumors received the maximum dose of 8 wafers (61.6 mg carmustine). Six patients had the wafers removed because of a serious AE (SAE); in 2 of those patients, the investigator believed that the SAE was possibly, or probably, related to the chemowafers, and these patients remained in the study.

The AEs reflected the morbidity of the primary cancer and the side effects of systemic therapy (eg, chemotherapy, radiation therapy, paraneoplastic syndromes, etc) (Table 2). SAEs (including deaths) were reported for 40 of 59 patients. Six patients had a total of 10 SAEs that were considered possibly related to the chemowafers (Table 3). None were fatal, and all resolved with appropriate surgical or medical management; 2 SAEs (a chemowafer-related infection and a wound infection) required removal of the wafers, and 1 SAE (the aforementioned chemowafer-related infection) had permanent sequelae. Nine patients died during the study, including 1 neurologic death and 8 deaths related to the extracranial, primary cancer.

**Table 2:** Incidence of Treatment-Emergent Adverse Events in ≥5% of Patients: Treated Population (n = 59)

Variable	No. of Patients (%)
No. with ≥1 treatment-emergent adverse event	53 (90)
Hematologic disorders	5 (9)
Anemia	3 (5)
Cardiac disorders	5 (9)
Bradycardia	3 (5)
Eye disorders	5 (9)
Vision blurred	3 (5)
Gastrointestinal disorders	21 (36)
Constipation	9 (15)
Nausea	9 (15)
Vomiting	4 (7)
General disorders and administration site conditions	25 (42)
Asthenia	7 (12)
Fatigue	13 (22)
Edema peripheral	6 (10)
Pyrexia	5 (9)
Infections	15 (25)
Candidiasis	3 (5)
Oral candidiasis	3 (5)
Metabolic disorders	23 (39)
Anorexia	3 (5)
Dehydration	4 (7)
Failure to thrive	3 (5)
Hyperglycemia	17 (29)
Musculoskeletal disorders	10 (17)
Muscular weakness	6 (10)
Neoplasms: Benign, malignant, and unspecified	5 (9)
Malignant neoplasm progression	3 (5)
Neurologic disorders	33 (56)
Aphasia	4 (7)
Seizure	6 (10)
Headache	13 (22)
Lethargy	3 (5)
Psychiatric disorders	12 (20)
Anxiety	4 (7)
Confusional state	3 (5)
Insomnia	3 (5)
Respiratory disorders	16 (27)
Dyspnea	6 (10)
Cutaneous disorders	11 (19)
Drug-induced rash	4 (7)
Vascular disorders	10 (17)
Deep vein thrombosis	6 (10)
Hypertension	4 (7)

**Table 3:** Serious Adverse Events

Patient No.	Serious Adverse Event	Severity	Related to Wafers?	Outcome
1	Intracranial hypotension	Severe	Probably	Resolved
	Soft tissue necrosis	Severe	Probably	Resolved
2	Chemowafer-related infection	Severe	Probably	Resolved with sequelae (wafers removed)
3	Wound infection	Severe	Probably	Resolved (wafers removed)
4	Asthenia	Moderate	Possibly	Resolved
	Dehydration	Moderate	Possibly	Resolved
	Convulsion	Moderate	Possibly	Resolved
5	Brain abscess	Severe	Possibly	Resolved
6	Mental status change	Moderate	Possibly	Resolved
	Convulsion	Severe	Possibly	Resolved

## Discussion

Our findings add to the growing body of evidence supporting intensive local and multifocal treatment for brain metastases^12^,^30^,^32^–^34^ with sparing of the surrounding white matter fibers, vessels, and myelin in an effort to preserve NCF.^35^ Neurocognitive testing provides a quantitative tool for monitoring the effects of therapy and developing tailored therapies to enhance functional well being.^36^ This study is significant because it demonstrates that: 1) the “cognitive trajectory” after craniotomy is one of preserved or improved NCF for the majority of patients, and 2) NCF can be improved or preserved while achieving acceptable rates of LC with a strategy of resection plus chemowafer, while deferring WBRT. Such an approach may be particularly appropriate when surgery is clinically indicated for a larger tumor (2-3 cm in greatest dimension), a symptomatic metastasis, or a lesion requiring tissue diagnosis.^37^

Brain metastases represent an increasing health priority because of the prolonged survival of cancer patients, the inability of some novel cancer therapies to reach the CNS, and enhanced detection using advanced neuroimaging.^2^,^28^ Thus, as the outlook for meaningful survival has increased with improved systemic therapy, the goals of brain metastasis therapy are shifting from short-term palliation to prolonged control of intracerebral tumor growth while maintaining/improving neurologic and functional status and avoiding the potential toxicity of WBRT.^12^,^18^,^19^,^32^,^33^,^37^,^38^ Indeed, results from the European Organization for Research and Treatment of Cancer (EORTC) 22952-26001 trial (surgery/SRS then WBRT or surveillance) support withholding the upfront use of WBRT, thereby improving functional outcomes, without compromising survival.^12^,^32^

The current results suggest that deferring WBRT while using upfront surgery and carmustine wafers can preserve NCF and provide acceptable local cancer control rates, similar to surgery and WBRT.^7^,^12^ We observed a global NCF decline in only 1 patient (2%), which is markedly superior to WBRT.^12^,^29^,^30^,^32^ NCF was preserved in all 3 domains in 65% of patients. In addition, NCF improvements were observed in >40% of patients for each individual domain, especially executive function and memory. The improvement may be caused by a reduction of perilesional vasogenic edema or the neuroplasticity of white matter fiber tracts. These findings compare favorably to a recent study of SRS with/without WBRT, in which memory and learning declined in 24% of patients who underwent SRS alone and in 52% of those who underwent SRS with WBRT.^30^

For patients with brain metastases, NCF scores correlate with tumor volume and predict survival^32^; >90% of patients with brain metastases demonstrate impairment of ≥1 neurocognitive tests at baseline,^32^,^36^ which can be improved with surgery.^37^ The current study demonstrates a sustained improvement in NCF after surgery and indicates that carmustine chemowafers do not adversely affect NCF.

The 28% local recurrence rate reported here is comparable to the previously reported rates for SRS alone^32^ and for surgery with WBRT^7^ and is generally superior to the rates reported from studies of surgery alone (without wafer).^7^,^32^ The addition of WBRT to SRS increases LC rates^13^,^32^ up to 97% for low-volume metastases.^39^ Previously, WBRT combined with surgery plus chemowafers produced complete (100%) LC.^25^ Because local recurrence rates are a function of both tumor volume and surgical technique,^40^,^41^ the extent to which local efficacy is attributable to advances in neurosurgical technology^40^,^41^ versus the effect of the carmustine wafer^42^ cannot be determined without a controlled comparative trial.

There were 9 deaths during the 12-month follow-up period, and 5 of those deaths occurred >300 days after surgery. It is noteworthy that only 1 patient (2%) died because of neurologic disease progression. In the EORTC study of surgery/SRS plus WBRT, neurologic death occurred in 28% of patients who received WBRT compared with 44% of patients who were managed with surveillance, although overall survival was unchanged.^32^ The low neurologic mortality rate compares favorably with patients who underwent SRS alone, reported as 12%.^33^

The toxicity and AEs reported here are largely because of the systemic cancer and concurrent therapies. Because the chemowafers release carmustine locally, there is no detectable level of chemotherapy in the bloodstream, avoiding many of the systemic toxicities.^20^–^22^ It is known that chemowafers carry neurosurgical risks of cerebral edema, wound dehiscence, or seizures,^20^,^21^ which are reduced by the judicious use of steroids, water-tight dural closures, and anticonvulsant medications.^20^,^21^,^25^ In our prior study of chemowafers, surgery, and WBRT, we observed seizures in 2 patients (8%), but no other toxicities.^25^

The current study has limitations. First, this was not a randomized, controlled trial. The specific benefits/toxicities of surgery, chemotherapy, SRS, and avoidance of WBRT were compared with historic controls. Placebo-controlled trials using chemowafers, which have proven efficacy in high-grade gliomas,^20^,^22^ are problematic to design for single and oligometastatic brain cancer, for which there are multiple existing options (eg, surgery alone, SRS alone, resection plus SRS, resection plus WBRT) and deeply held biases.^18^ Second, single-arm studies of NCF in patients with brain metastases are difficult to interpret because of numerous confounding variables, including selection bias.^43^ In the current study, there was a selection bias, because our patients had an RPA classification of 1 or 2, a favorable KPS, and a prognosis that favored undergoing craniotomy. Nevertheless, each patient acted as their own control, so an immediate postoperative and sustained improvement in NCF is noteworthy and novel. Current approaches to the treatment of brain metastases must balance local and distant control in the CNS with neurocognitive preservation. The current study demonstrates that the strategy of neurosurgical resection plus chemowafers provides local cancer control comparable to that provided by radiation therapy. Furthermore, the results indicate a benefit in NCF from withholding WBRT and treating limited brain metastases in a targeted manner. The range of options for patients with brain metastases also will be increased as the novel, pathway-specific, targeted agents^44^ are evaluated. The preserved and often improved NCF observed using resection and local carmustine wafers, as well as the known pharmacokinetics of carmustine,^20^,^21^,^42^ suggest that this strategy can be used without injury to white matter fiber tracts that affect NCF.^45^
